# A semi-automated algorithm for image analysis of respiratory organoids

**DOI:** 10.1371/journal.pcbi.1013589

**Published:** 2025-10-27

**Authors:** Anna Demchenko, Maxim Balyasin, Elena Kondratyeva, Tatiana Kyian, Alyona Sorokina, Marina Loguinova, Svetlana Smirnikhina

**Affiliations:** 1 Research Centre for Medical Genetics, Moscow, Russian Federation; 2 Peoples’ Friendship University of Russia, Moscow, Russian Federation; 3 Endocrinology Research Center, Moscow, Russian Federation; University of Zurich Faculty of Mathematics and Science: Universitat Zurich Mathematisch-Naturwissenschaftliche Fakultat, SWITZERLAND

## Abstract

Respiratory organoids have emerged as a powerful *in vitro* model for studying respiratory diseases and drug discovery. However, the high-throughput analysis of organoid images remains a challenge due to the lack of automated and accurate segmentation tools. This study presents a semi-automatic algorithm for image analysis of respiratory organoids (nasal and lung organoids), employing the U-Net architecture and CellProfiler for organoids segmentation. The algorithm processes bright-field images acquired through z-stack fusion and stitching. The model demonstrated a high level of accuracy, as evidenced by an intersection-over-union metric (IoU) of 0.8856, F1-score = 0.937 and an accuracy of 0.9953. Applied to forskolin-induced swelling assays of lung organoids, the algorithm successfully quantified functional differences in Cystic Fibrosis Transmembrane conductance Regulator (CFTR)-channel activity between healthy donor and cystic fibrosis patient-derived organoids, without fluorescent dyes. Additionally, an open-source dataset of 827 annotated respiratory organoid images was provided to facilitate further research. Our results demonstrate the potential of deep learning to enhance the efficiency and accuracy of high-throughput respiratory organoid analysis for future therapeutic screening applications.

## Author summary

In this study, we developed a semi-automated tool to analyze images of respiratory organoids—3D cell structures that mimic the human respiratory system. These organoids are vital for studying diseases like cystic fibrosis and testing potential drugs, but manually analyzing their images is time-consuming and prone to errors. Our tool uses artificial intelligence (AI) to quickly and accurately measure organoid size and shape from bright-field microscope images, eliminating the need for fluorescent dyes that can harm cells. We trained our AI model on a publicly shared dataset of 827 annotated organoid images, achieving high accuracy in detecting and quantifying organoids. When applied to cystic fibrosis research, the tool successfully measured differences in organoid swelling (forskolin-induced swelling - a key test for drug response) between healthy and patient-derived samples. By making our dataset and method openly available, we hope to support further research into respiratory diseases. Our work bridges the gap between complex lab techniques and practical applications, offering a faster, more reliable way to study human health and disease.

## Introduction

Organoids are three-dimensional cell cultures that recapitulate the structure and function of organs, making them established models for *in vitro* studies of drug discovery and disease mechanisms [1–3]. Organoids of the respiratory tract, such as nasal and lung organoids (NOs and LOs, respectively), have emerged as particularly useful models for studying various respiratory conditions including chronic obstructive pulmonary disease, cystic fibrosis (CF), viral infections, idiopathic pulmonary fibrosis (IPF), and other pulmonary diseases [4–7]. Respiratory organoids can be derived either from biopsy material [7–9], or by directed differentiation from human induced pluripotent stem cells (hiPSCs) [10,11]. Obtaining organoids from hiPSCs offers several advantages, including scalability (iPSCs provide an unlimited cell source), less invasive procedures using accessible somatic cells (e.g., skin or blood), and a more standardized and reproducible cell platform [12,13].

CF is one of the most common monogenic hereditary diseases which occurs due to mutations in the *CFTR* gene. The number of patients in the world exceeds 100 thousand people (about 4 thousand live in Russia [14], more than 33 thousand are registered in the USA [15], more than 56 thousand - in Europe [16]), of whom from 52% to 85.5% have at least one copy of F508del variant (https://www.cff.org/medical-professionals/patient-registry). The *CFTR* gene encodes a chloride ion channel on the apical surface of epithelial cells. The homozygous F508del variant disrupts protein folding and transport to the membrane, impairing chloride and sodium ion transport [17]. In CF it is relevant to assess the functional activity of CFTR-channel using the forskolin-induced swelling (FIS) test, the effect of which is based on activation of CFTR-channel, which results in the influx of fluid into the lumen of the organoid and swelling of the organoid is observed. Currently, FIS on intestinal organoids has found widespread use in the selection of targeted therapy for patients with CF (drug-induced swelling, DIS) [18], however, respiratory tract organoids such as NOs and LOs are also beginning to be used as they are a more relevant disease model [19]. Organoid functional analysis generates hundreds or thousands of organoid images that need to be analyzed to assess the efficacy of therapy. Morphometric image analysis software such as ImageJ and CellProfiler are commonly used to analyze images from FIS or DIS assays. However, these approaches require manual parameter selection and settings adjustment [20,21], which makes them labour-intensive and potentially subject to user bias.

For accurate, automated and high-throughput analysis, it is necessary to automate the process of organoid image analysis. Recent advances in AI, particularly deep learning models such as convolutional neural networks (CNNs), offer promising solutions for automated organoid image analysis [22]. For instance, a recent comprehensive benchmark study by Cicceri et al. demonstrated the superior effectiveness of Deep Learning models, including vision transformers, over traditional machine learning methods in capturing subtle morphological differences for the classification of intestinal organoids, reinforcing the critical need for automated and scalable analysis frameworks in organoid research [23]. Table 1 provides an overview of the main organoid imaging programs currently available. These programs typically work with images captured in phase contrast or bright-field (BF), eliminating the need for dyes to visualize organoids. This approach reduces manipulation steps, avoids the cytotoxic effects of dyes and UV exposure on organoids, and decreases the time required for FIS analysis. For segmentation and object detection tasks, there are several popular model architectures such as R-CNN, Mask R-CNN, YOLOX and their variations. A model architecture specialized for image segmentation tasks is U-Net, which shows higher accuracy and is often used in morphometric algorithms and software such as StarDist, Cellpose and DeepMIB [24–27]. As shown in Table 1, three programs (OrganoID, OrganoLabeler, OrgaExtractor) are based on the U-Net algorithm, while the remaining programs utilize other CNN architectures. U-Net is based on semantic segmentation, dividing the image into semantic classes (e.g., background, live cells, dead cells) without separating individual objects within a class. This approach is simpler compared to instance segmentation methods like Mask R-CNN, which often show lower accuracy in semantic segmentation tasks [28,29]. The potential of organoid-based models extends beyond mere image analysis into the emerging field of ‘organoid intelligence’, which aims to harness the computational potential of brain organoids combined with AI [30]. This interdisciplinary approach, leveraging microfluidics, electrophysiology, and deep learning, holds transformative potential for biocomputing and disease modeling, as highlighted in recent perspectives. However, this also introduces complex ethical and regulatory considerations that must be addressed alongside technical development. For LOs specifically, the Deep-LUMEN program can classify the polarity of spheroids from BF images, but it is limited to spheroids obtained from adenocarcinomic human alveolar basal epithelial cells (A549 cells line) [31]. Bian et al. described an algorithm trained on images of mouse liver organoids and alveolar organoids [32]. However, the program uses a method of determining object coordinates using a bounding box, which does not allow accurate object segmentation. Two automatic programs trained on intestinal organoids and used to analyze organoid morphology after forskolin exposure are described [33,34], but both programs also define the bounding box of organoids and do not allow to define the exact boundaries of all organoids in the image.

**Table 1:** Overview of the main programs for organoid image analysis.

Program	Algorithm	Annotation and tracking of organoids	Input images	Objects	Accuracy of algorithm	Program availability	Dataset	References
OrganoID	U-Net	Both	BF and phase-contrast images	Mouse small intestinal organoids	Tracking maintained over 89% accuracy. Organoid counts agreed with a CCC of 0.95. IoU = 0.74	Open-source	66 images	[35]
OrgaQuant	R-CNN and Faster R-CNN	Both	BF images	Human intestinal organoids	mAP of 80%	Open-source	1750 images	[36]
OrganoSeg	MATLAB using the Image Processing Toolbox	Annotation	BF, phase-contrast, and differential-interference contrast images	Breast-cancer spheroid, colon and colorectal cancer organoid	Sensitivity 92% Positive predictive value 96%	Open-source	N/A	[37]
OrgaSegment	MASK-R-CNN	Both	BF images	Intestinal organoids	N/A	Open-source	231 images	[38]
OrganoIDNet	DL algorithm (trained a StarDist model n using custom dataset)	Both	BF images	Human pancreatic ductal adenocarcinoma organoids	nFP did not exceed 18.5%, and nFN counts were no more than 16.4%	Open-source	180 images	[39,40]
OrganoLabeler	U-Net	Annotation	BF images	Embryoid body (EB) and brain organoid (BO)	IoU: EB – 0.71 BO – 0.91	Open-source	165 images of EB, 133 images of BO	[41]
OrgaExtractor	U-Net	Annotation	BF images	Colon organoids	Sensitivity 83.8% Specificity 76.9% Accuracy 81.3%	Open-source	248 images	[42]
Deep-LUMEN	Faster R-CNN ResNet101 with data augmentation	Both	BF images	Lung spheroid (A549 cells line)	mAP 83%	Open-source	4000 images	[31]
Deep-Orga	Lightweight model YOLOX	Annotation	BF images	Intestinal organoid	mAP 72.2%	Open-source	1750 images	[43]
D-CryptO	XCeption	Both	BF images	Colon organoid	Accuracy: for opacity – 98%, for budding – 90.87%	N/A	The initial dataset comprised 35 images, which were later expanded	[33]
OrBITS	CNN	Both	BF images	Lung and pancreatic cancer organoids	N/A	Available to researchers upon reasonable request and discussion with the corresponding author	N/A	[44]
A deep learning model for detection and tracking in high-throughput images of organoid	Several DL-based: Faster_R-CNN, R-CNN, Free_anchor, FoveaBox and other	Both	Phase-contrast images	Mice liver organoids and alveolar organoids	mAP 80.9%	Open-source	75 images	[32]

Abbreviations: BF - bright-field, CCC - concordance correlation coefficient, IoU - intersection-over-union metric, CNN - Convolutional Neural Network, R-CNN - Region Convolutional Neural Network, mAP - mean Average Precision, DL - deep learning, N/A - not available, nFP - normalized false positive, nFN - normalized false negative.

In this work, we describe a new semi-automatic algorithm for analyzing NOs and hiPSCs-derived lung organoids (hiLOs) and it is application in the analysis of FIS. While several organoid image analysis tools exist (Table 1), a significant gap remains for solutions specifically tailored to respiratory organoids derived from both primary tissues and hiPSCs. Most available programs are trained on intestinal organoids [33–38], rely on bounding boxes that preclude precise morphological analysis [32,33], or are not designed for high-throughput z-stack imaging of entire wells—a critical requirement for drug screening pipelines. In our algorithm, we propose to acquire images of the whole drop with organoids and capture images along the z-axis, followed by horizontal and vertical stitching and focus stacking. We selected the U-Net architecture for image segmentation and employed CellProfiler to separate organoids into individual objects. Additionally, we provide an open-source dataset that includes stitched BF images with manually labeled binary masks after focus stacking, addressing the current lack of publicly available datasets for respiratory organoids.

## Results

### Algorithm for image analysis

**Fig 1:**
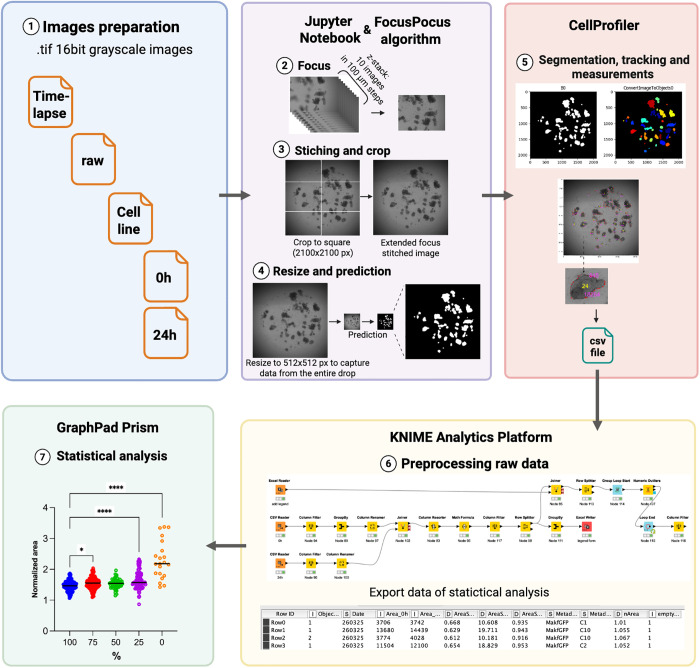
Schematic representation of semi-automated algorithm for organoids analyzing. The pipeline begins with the acquisition of raw bright-field z-stack images from multiple fields of view (1). Then images are processed to generate a single, sharp EFSI for each well, cropped (2-3) and preprocessed for optimal segmentation by a trained U-Net convolutional neural network, which produces a primary binary mask (4). This mask is subsequently refined by a CellProfiler pipeline that separates clustered organoids, filters out artifacts based on size and shape, and performs instance segmentation to identify individual organoids, then extracts quantitative morphological parameters for each object (5) including unique ID assignment for temporal tracking. For time-course experiments, CellProfiler tracks organoids across time points using position-based matching applied in reverse chronological order to mitigate occlusion errors. Finally, the extracted data is integrated and analyzed in a KNIME workflow to calculate key metrics (6) and generate consolidated tables for statistical analysis (7). This figure was created using the Biorender application.

A comprehensive semi-automated algorithm was developed for analyzing morphological characteristics of respiratory organoids (Fig 1). The system integrates multiple computational platforms, beginning with a Jupyter Notebook containing code that prepares source images for model application, performs image processing, and saves both stitched images and corresponding masks. The algorithm’s workflow processes raw microscopic images captured using a 4 × objective lens across various focal planes (10 focus levels with 100 μm increments) from multiple fields of view (FOV) for each well. The initial processing phase implements z-stack fusion to generate sharp, in-focus organoid images, followed by x-y axis stitching to create extended focus stitched image (EFSI). These images undergo additional preprocessing including cropping, resizing, and prediction operations to optimize them for subsequent analysis.

Following image preparation, the U-Net model performs semantic segmentation to generate primary binary masks distinguishing organoid pixels from background. These masks are subsequently processed through a customized CellProfiler pipeline that executes instance segmentation to separate touching organoids into individual objects using watershed algorithms, applies morphological filtering to remove artifacts based on size and circularity criteria, and enables temporal tracking by assigning unique identifiers to each organoid and matching them across time points via position-based algorithms. This stage incorporates primary filtration protocols to eliminate artifacts and objects that don’t meet size criteria (e.g., extremely small particles or atypical structures).

CellProfiler then extracts per-organoid 2D morphometrics from the binary masks, including area, perimeter, centroid, axes and orientation, eccentricity, form factor (4πA/P²), solidity, extent, compactness, Euler number, radii and Feret diameters. These measurements are computed by CellProfiler’s MeasureObjectSizeShape module on the binary masks. A distinctive feature of our approach is the organoid tracking methodology, which is position-based and proceeds in reverse chronological order—starting from later time points and working backward. This reverse tracking strategy is implemented because organoids typically increase in size over time and larger, later-stage organoids would otherwise obscure their smaller initial states in the tracking process.

The resultant analysis tables are exported to a KNIME workbook for advanced data integration, including compilation of organoid data across different time points (e.g., “0 h” and “24 h”), calculation of size increments, and generation of visualization elements necessary for subsequent statistical analysis. These processed datasets are formatted for compatibility with statistical analysis software such as GraphPad Prism or equivalent platforms. This algorithmic approach ensures automated and reproducible processing of organoid images and generates consolidated analytical tables suitable for comprehensive biostatistical analysis.

### Model evaluation

**Fig 2:**
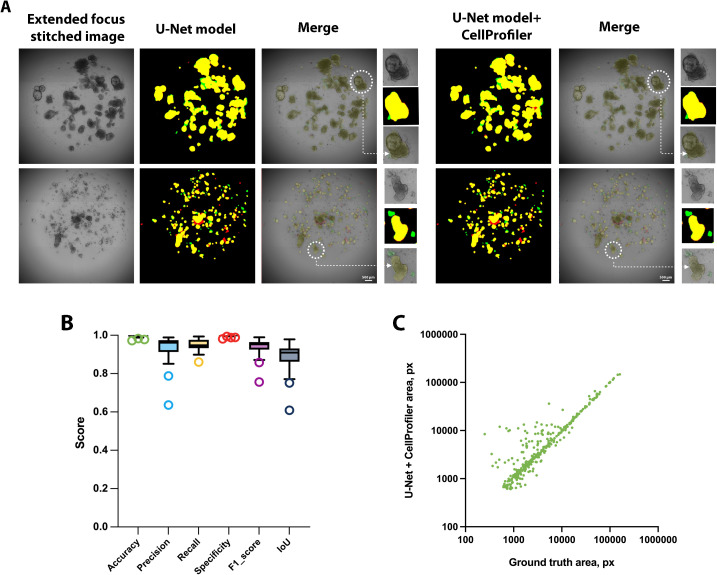
Performance of the model on the validation dataset of EFSI. (A) Binary masks predicted by the U-Net model and with CellProfiler post-processing (U-Net model+ CellProfiler), with color-coding: yellow - true positives, green - false negatives, red - false positives. (B) Performance metrics of U-Net model output with CellProfiler post-processing on validation set excluding images with minimal annotations (<5000 px) containing primarily background. (C) Correlation plot of individual object sizes from manual annotations (ground truth annotations) versus model (U-Net model + CellProfiler) predictions in the validation dataset excluding images with minimal annotations (<5000 px), (R² = 0.9872, p < 0.0001).

The U-Net model was trained on 703 images (85% of the total dataset), with concurrent validation performed on 83 images (10%), followed by final performance assessment on a test set of 41 images (5% of the dataset). Initially, manual segmentation masks were generated by two independent annotators. Inter-annotator agreement (per-image F1 and IoU) was F1 = 0.9086 ± 0.1048; IoU = 0.8461 ± 0.1456. The evaluation ground truth was defined as the intersection (AND operation) of the two annotations to form a consensus mask. Consequently, the model demonstrated high efficiency in organoid segmentation (Fig 2A) across the test set images which includes all types of organoids presented in Table 2. The raw model outputs achieved mean metric values of IoU (calculated at the pixel level for the organoid class) = 0.7007, Accuracy = 0.9967, and F1 = 0.7613. Post-processing of the model’s binary mask outputs using CellProfiler software (Broad Institute of MIT and Harvard, USA) [45] allowed for slight improvements in performance metrics: IoU = 0.7068, Accuracy = 0.9968, and F1 score = 0.7574. Notably, the validation dataset included 14 images containing ≤5000 labeled px (0.1% of the image) without organoids, consisting only of background, bubbles, or dead organoids. When evaluating the subset of 27 images containing viable organoids, the raw model outputs achieved substantially higher metrics: IoU = 0.879, Accuracy = 0.9952, and F1 = 0.9334. For the CellProfiler-processed masks, the values were: IoU = 0.8856, Accuracy = 0.9953, and F1 = 0.9373 (Fig 2B). The comprehensive model performance parameters are presented in Table A in S1 Text. Correlation analysis for individual organoids between the U-Net model-predicted masks (post-processed with CellProfiler) and the original ground truth annotations revealed a strong correlation coefficient of R² = 0.9872 (p < 0.0001, Fig 2C). Since processing the masks obtained by U-Net model using CellProfiler slightly increases the resulting power, we use this approach to subsequent analyze the organoid images. Moreover, the performance of our model was benchmarked against two established methods: the classical object segmentation pipeline, CellProfiler, and the widely used ilastik pixel classifier (European Molecular Biology Laboratory), an accessible machine learning-based solution for object segmentation [46]. The CellProfiler pipeline for generating organoids binary masks has been made available in the dataset repository https://osf.io/4savy/?view_only=2163a86b20a5468989c041536752b19e. The ilastik software was trained in a manner analogous to the U-Net model, utilizing the same set of images. Similarly, the parameters for the minimum cross-entropy thresholding method in CellProfiler were manually optimized using the same training dataset. On a test set of 41 images, CellProfiler achieved IoU = 0.4039, Accuracy = 0.975, and F1-score = 0.4989, while ilastik showed IoU = 0.3984, Accuracy = 0.9806, and F1-score = 0.4988 (Fig B in S1 Text). Additionally, for a subset of 27 images containing viable organoids, CellProfiler achieved IoU = 0.5818, Accuracy = 0.9775, and F1-score = 0.7071, while ilastik showed IoU = 0.5665, Accuracy = 0.9774, and F1-score = 0.6951. Therefore, our model provides more accurate and spatially coherent segmentations than CellProfiler and ilastik, as reflected by consistently higher IoU and F1 scores.

**Table 2:** List of organoids lines used in the research.

Donor	Origin	Organoid line	*CFTR* genotype	References on hiPSC line
1	hiPSCs-derived	hiLOs P1L5	F508del/F508del	[58]
2		hiLOs P5L5	F508del/F508del	[59]
3		hiLOs P7L2	F508del/F508del	[60]
4		hiLOs Mak-f	WT/WT	[61]
5	Primary	hNOs 35	WT/WT	–
6		hNOs 43	WT/WT	–

### Application of the model to the detection of respiratory organoids

**Fig 3:**
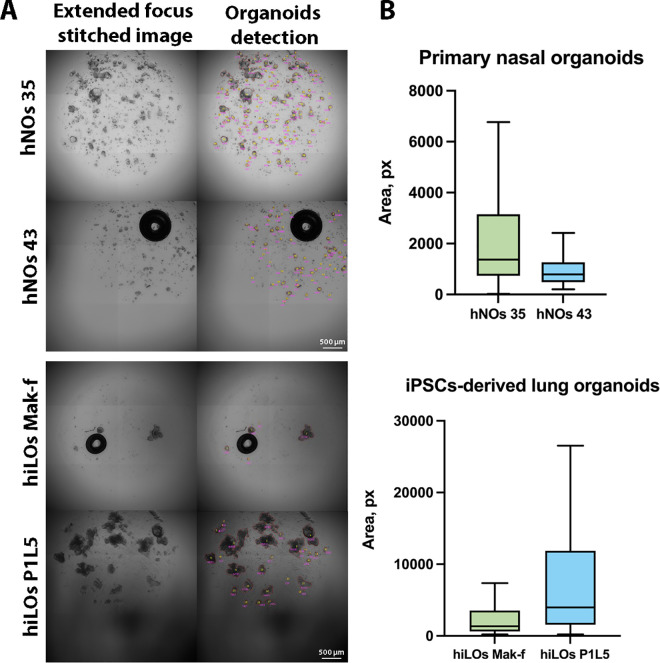
Performance of the U-Net model with CellProfiler post-processing for detection and area determination of respiratory organoids on new data. (A) Detection of respiratory organoids of different size, morphology, with the presence of bubbles and irregular illumination. (B) Distribution of individual organoid areas showing distinct size profiles. Data are presented as median (Q1,Q3).

The U-Net model with CellProfiler post-processing was applied to the detection of new data of respiratory organoids and compared respiratory organoid sizes. Primary hNOs derived from two donors (hNOs 35 and hNOs 43) and hiLOs derived from two donors (hiLOs P1L5 and hiLOs Mak-f) were used for this research. As can be seen in Fig 3A, respiratory organoids can vary in size and morphology. Bubbles may be present within the droplets containing the organoids, and the illumination may be irregular during image acquisition. The developed semi- automated algorithm successfully copes with these issues, as it does not encircle the bubbles and can detect organoids of different sizes.

Analysis of respiratory organoid area using the U-Net model with CellProfiler post-processing revealed significant differences in organoid size (Fig 3B). Median hNOs area values were 1369 px or 5476 µm^2^ (Q1,Q3: 736–3150 px, n = 1703 organoids) for hNOs 35 and 792 px 3168 µm^2^ (Q1,Q3: 483–1261 px, n = 3418 organoids) for hNOs 43. For hiPSCs-derived organoids shown that hiLOs Mak-f (healthy donor) had a mean area of 1341 px or 5364 µm^2^ (Q1,Q3: 620–3538 px, n = 103 organoids), while hiLOs P1L5 (from a CF patient) were larger with a mean area of 3982 px or 15928 µm^2^ (Q1,Q3: 1558–11872 px, n = 428 organoids). These quantitative measurements, performed by semi-automated analysis, demonstrate the ability of the model to detect and characterize significant morphological differences between organoids.

### Application of the model to the forskolin-induced swelling assay

**Fig 4:**
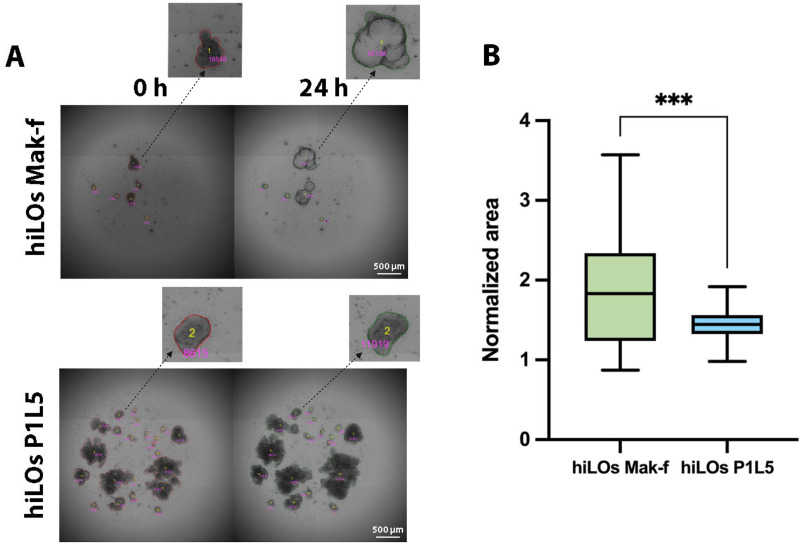
FIS image analysis using the semi-automatic algorithm. (A) Representative EFSI of hiLOs with organoid detection, taken before and after 24 h of stimulation with 10 µM forskolin. Organoid identifiers (IDs) are annotated in yellow, while corresponding area measurements (in pixels) are indicated in pink. (B) Quantification of the hiLOs swelling area at 24 h, normalized to the value at 0 h. Data are presented as a box-and-whisker plot by Tukey methods, *** - p < 0.001.

The U-Net model with CellProfiler post-processing was applied to analyze the FIS assay of hiLOs from healthy donor Mak-f and from CF patient (homozygous for F508del in *CFTR*) P1L5 (Fig 4A). The hiLOs P1L5 demonstrated reduced swelling capacity in the FIS assay, the swelling ratio at 24 h compared to baseline (0 h) was significantly greater in healthy organoids (Welch test, p = 0.0006) (Fig 4B). The hiLOs Mak-f showed a swelling ratio of 1.831-fold change (Q1,Q3: 1.238 to 2.34, n = 44 organoids), while the hiLOs P1L5 exhibited a more modest increase of 1.445-fold change (Q1,Q3: 1.323 to 1.563, n = 228 organoids), which confirms functional differences in CFTR-channel activity between healthy and patient-derived hiLOs. The semi-automated analysis effectively quantifies both morphological differences and functional responses, highlighting the model’s utility in distinguishing between healthy and disease phenotypes. Thus, we have shown the possibility of FIS analysis without fluorescent dye, using semi-automatic image analysis, which should significantly increase the throughput of the method and make it more convenient and faster in the screening of drugs aimed at the treatment of CF.

## Discussion

In this study, we present a deep learning algorithm that is capable of automatically generating high-resolution extended focus stitched images and analyzing organoid images in the bright-field based on the U-Net model. The availability of an automated or semi-automated program facilitates the evaluation of large image data, a process that is essential for high-throughput organoid screening, where manual analysis is not possible. Respiratory organoids have been extensively used for respiratory disease modeling [47–49] and drug testing [19,50,51]. However, the development of algorithms that are trained on images of primary and induced respiratory organoids for automated analysis has not been previously published. As organoids are three-dimensional objects which reside in the thickness of the extracellular matrix (e.g., Matrigel), it is not possible to detect all organoids in focus. Therefore, it is reasonable to apply z-stack technology when imaging organoids in order to capture all organoids. It was found that only one program was capable of this function [32]. Compared to most existing algorithms, our algorithm is designed to work with images from high-throughput analysis, where images are acquired automatically from the entire well and with 10 levels of focus (1000 µm thickness in 100 µm steps) without loss of information and manual FOV selection. The employment of semantic segmentation within the algorithm, as compared to the use of bounding boxes, enables the analysis of organoid morphology, which is a pivotal aspect in the evaluation of swelling in FIS and DIS tests, as well as the assessment of organoids viability. We showed that our algorithm is able to accurately (IoU = 0.8856, Accuracy = 0.9953, F1 = 0.937) detect hiLOs and NOs of different shapes (large, small, round, spherical and branching). An IoU greater than 0.5 is generally considered to reflect a good prediction [52,53]. It should be noted that while the accuracy of 0.9953 appears exceptionally high in our study, this metric is profoundly misleading due to the extreme class imbalance between “background” and “organoids” inherent in our image data. Therefore, accuracy is not a relevant performance metric for our task, and we primarily rely on the IoU and F1-score, which are specifically designed to be robust to such class imbalance. Our obtained values are not inferior, but even superior to the accuracy of such algorithms as OrganoID (IoU = 0.74) and OrgaExtractor (Accuracy = 81.3%) [35,41]. However, direct cross-study performance comparison is limited by domain shift—models trained under different imaging conditions (resolution, illumination, microscopy systems) and on different organoid types typically generalize poorly without fine-tuning. As expected, the proposed U‑Net–based segmentation model achieves higher accuracy than the CellProfiler and the standard ilastik pixel classifier, because CNNs learn multi‑scale, context‑aware representations that capture spatial structure and object boundaries rather than classifying pixels independently. A limitation of the present algorithm is that it was evaluated using data from a single microscope type, which may limit its applicability to other laboratories. The dependence on the Lionheart FX microscope can be addressed by pre-training the model on data from alternative devices. An additional limitation is the specificity of the experimental design, covering both the methodology for obtaining organoids and the specifics of their morphological characteristics during culturing and FIS analysis. Nevertheless, the proposed methodological approach provides researchers with the possibility of adapting and additional training of the model to solve specific problems within their own laboratory conditions.

We evaluated the hiLOs response in a FIS assay without fluorescent dye using the developed algorithm for the first time. The automation and simplification of the FIS and DIS assay has the potential to accelerate the process of CFTR modulator screening for patients with rare mutations. Previously, the protocol for performing FIS assay always included the use of calcein green [54,55]. However, as has been repeatedly discussed, the use of fluorescent dyes can be associated with cytotoxic effects on cells [56] and also diffuse unevenly through the extracellular matrix. In case of prolonged FIS assay (12–24 h), which is typical for respiratory organoids, the dye is washed out and it is necessary to reapply the dye, a process that extends the experiment and has the potential to displace the droplets of extracellular matrix in the well due to additional mechanical manipulations.

The availability of open-source datasets is necessary for training and testing of algorithms, and no such datasets were previously available for NOs and LOs. We provide an open-source respiratory organoids dataset including 827 organoid images, which will be useful for the development of high-throughput screening of respiratory diseases. The algorithm has been tested on only two types of organoids, but due to the fact that the training sample and the analysis are as diverse in morphology as possible, the algorithm can be adapted to analyze alveolar, bronchial, and other types of organoids.

To summarize, we described an algorithm for data acquisition and image analysis of respiratory organoids using convolutional neural networks U-Net. This algorithm is capable of detecting organoids with a high degree of accuracy for subsequent analysis of the obtained data on organoid morphometry. The study demonstrates the application of the algorithm in FIS assay, which allows to optimize the process of both the analysis itself (due to the work of the algorithm with BF images and the absence of fluorescent dye) and the processing of the obtained images to increase the throughput of the analysis. Semi-automated pipeline shows significant potential for clinical integration, particularly in personalized medicine platforms for CF. By enabling high-throughput, dye-free functional analysis of patient-derived organoids, this approach could accelerate the selection of effective CFTR modulator combinations for individuals with rare mutations, ultimately supporting treatment personalization.

## Materials and methods

### Ethics statement

The study was approved by the Ethics Committee of the Research Centre for Medical Genetics (Moscow, Russia) and conducted in accordance with the provisions of the Declaration of Helsinki of 1975. Patients and healthy donors signed informed written consent forms as anonymous participants in the study and donors of biological materials.

### Cell and organoids cultures

Human NOs (hNOs) were generated from human nasal epithelial cells (hNECs) derived from two donors (Table 2). To obtain hNECs, brush biopsies of nasal epithelium were placed in a sterile tube containing a transport medium consisting of DMEM (PanEco, Russia), 10 µg/mL of fungin (InvivoGen, France), 100 u/mL of penicillin and 100 µg/mL of streptomycin (PanEco, Russia). The resulting biomaterial was centrifuged for 5 min at 150 × g and cultured on culture plates coated with Matrigel (Corning, USA) in basal cell media (BCM) consisting of PneumaCult-Ex Plus Medium (STEMCELL Technologies, Canada) with 1 µM A83-01 (STEMCELL Technologies, Canada), 1 μM DMH1 (Sigma Aldrich, USA), 0.2 μM Hydrocortisone (STEMCELL Technologies, Canada), and 100 × penicillin-streptomycin (PanEco, Russia). For generation of hNOs, hNECs were harvested with the 0.25% Trypsin-EDTA solution (PanEco, Russia), counted using a Countess II FL Automated Cell Counter (Thermo Fisher Scientific, USA), and centrifuged at 150 × g for 5 min. The pellet was resuspended in undiluted cold Matrigel at a concentration of 1,000 cells/μL and replated in 10 μL drops into the wells of a 48-well plate (Corning, USA). The drops were allowed to solidify for 40 min in an incubator, after which the medium for nasal organoids were added. On day 7 after hNOs assembly, organoids were passage into a 96-well plate with a 3 μl drop volume in nasal organoid medium. Medium for nasal organoids consist of Serum-free differentiation medium (SFDM) with 250 ng/mL FGF2 (R&D Systems, USA), 100 ng/mL FGF10 (R&D Systems, USA), 50 nM dexamethasone (Sigma Aldrich, USA), 0.1 mM 8-bromo-cAMP (Sigma Aldrich, USA) and 0.1 mM 3-isobutyl-1-methylxanthine (Sigma Aldrich, USA). SFDM consist of 75% IMDM (Thermo Fisher Scientific, USA), 25% Ham’s F12 (PanEco, Russia), 100 × B-27 (Thermo Fisher Scientific, USA), 200 × N2 (PanEco, Russia), 0.05% bovine serum albumin solution (Sigma Aldrich, USA), 0.45 mM 1-thioglycerol (Sigma Aldrich, USA), 100 × GlutaMAX (Thermo Fisher Scientific, USA), 0.05 mg/mL L-ascorbic acid (Sigma Aldrich, USA) and 100 × penicillin-streptomycin. Characterization of hNECs and hNOs is presented in Fig A Text.

hiLOs were generated from induced basal cells (hiBCs) derived from hiPSCs using a protocol described previously [57]. Three hiBC lines of 3–5 passages obtained from three cystic fibrosis patients with homozygous F508del mutation in *CFTR* gene and one hiBC line from healthy donor were used (Table 2). hiBCs were cultured on culture plates coated with Matrigel in BCM medium. hiLOs derived from hiBCs were cultures into growth-factor reduced Matrigel and the medium for the hiLOs (lung medium) consisted of SFDM with 10 ng/mL FGF7 (R&D Systems, USA), 10 ng/mL FGF10, 10 ng/mL EGF (R&D Systems, USA) and 3 µM CHIR99021 (Tocris, UK). FIS assay was performed on day 7 after hiLOs assembly.

### Dataset creation and model training

To compile the dataset, we acquired images of 6 FOV from wells of a 96-well plate at different focal plane levels. Images were captured using a Lionheart FX automated imager (BioTek, USA) at 4 × magnification in the BF channel, with 2 images along the x-axis and 3 images along the y-axis. Each FOV contained 10 focus levels with 100 μm increments. The images were 1224 × 904 pixels (px) and saved in 16-bit.tif format. We processed the images using our developed FocusPocus algorithm, which generates EFSI through a simple z-stack focus algorithm followed by xy stitching. Subsequently, the edges were cropped to form a square, resulting in a single high-resolution EFSI with a pixel size of 2.0 μm/px and dimensions of 2100 × 2100 px. Ground truth masks were generated by two independent annotators blinded to model outputs using polygon tools in LabelMe software version 5.1.1 in strict accordance with written annotation guidelines, which required tracing the outer visible contour of each organoid, excluding apoptotic and dead organoids, vesicles, debris/particles, and single cells or small clusters under a minimum area threshold of 300 px², while also specifying that touching objects should not be separated and were to be annotated as a single instance. A total of 827 annotated organoid images and their binary masks were obtained. Inter-annotator agreement was computed per image (F1, IoU). The reference ground truth was obtained via pixel-wise Intersection (AND) of the two masks (unanimous consensus). The images were then downsampled to 512 × 512 px (downsample factor 4.1). The training images and their masks were saved in.png format.

To train a U-Net model for segmentation of BF organoid images, a sample of 827 manually labelled organoid images from two respiratory organoid cultures (4 hiLO lines and 2 hNO lines) was compiled. The total dataset was stratified into three subsamples: 703 images for training (85%), 83 validation (10%) and 41 tests (post-training model accuracy assessment, 5%). Before training, images were normalized by 1–99 percentile and contrast was improved by CLAHE (Contrast Limited Adaptive Histogram Equalization) with parameters clip limit = 1, tile size = 32. Training was performed in DeepMIB version 2.91 beta20 (University of Helsinki, Finland). Segmentation was performed using a CNN of U-Net architecture with 5 depth levels, filter size 3 and 32 filters. Activation layer was ReLu, segmentation layer is dice pixel classification. For training, 100% of the training dataset was augmented with reflection, rotation, shear, scale, blur, noise, brightness and contrast jitter. Solver was stochastic gradient descent with momentum with learning rate 0.01 and learning rate drop factor 0.5 every 75 epoch. Mini batch size was 16 images. The training lasted for 1000 epochs. The resulting model was evaluated using IoU, F1-Score and Accuracy metrics. The standard ilastik pixel classifier was selected for benchmarking and was trained on the identical dataset, such as the U-Net model. No data augmentation was performed, training on a standard set of features. All model training and inference were performed on a laptop equipped with an AMD Ryzen 7 7735HS CPU, 16 GB DDR5 RAM, and an NVIDIA GeForce RTX 4060 Laptop GPU, with a total training time of approximately 20 h. The full dataset, which allows reproduce the results of research, including the images and annotations, are publicly available at https://osf.io/4savy/?view_only=2163a86b20a5468989c041536752b19e under an MIT license.

### FIS assay and images accuration

To perform the FIS assay, hiLOs were passage into a 96-well plate with a 3 μl drop volume in lung organoid medium. On the first day of analysis, forskolin at a final concentration of 10 μM was added to the wells containing the organoids, then images were acquired on a Lionheart FX automated imager in the BF channel with a 4 × magnification as described in the section above. Then, after incubation for 24 h, images were acquired on a Lionheart FX Automated Microscope again.

### Statistical analysis

The statistical analysis and representation of the data were conducted using GraphPad Prism v.9.1.1. Descriptive statistics were calculated as the mean values with standard deviations (SD) as well as median values with interquartile ranges (Q1-Q3). For comparison of organoid sizes between groups, both parametric (Welch’s t-test) and non-parametric (Mann-Whitney U test) methods were applied depending on data distribution characteristics. In order to analyze the normalized swelling areas of hiLOs over time, a subset of hiLOs with area ≥1500 px and ≥0.8-fold change was selected, with 0 h of incubation set as a 1-fold increase. Statistical analysis was performed using a one-way analysis of variance (ANOVA) with post hoc Sidak tests. Correlation between parameters was assessed using Pearson’s correlation coefficient. The data were considered statistically significant at a p-value of less than 0.05.

## Supporting information

S1 Text **Table A.** Model performance metrics. Fig A. Characterisation of hNECs and hNOs (donor 4). A - Representative phase-contrast images of of hNECs on 1st passage and hNOs at 7 days of generation from hNECs. Scale bar, 200 μm. B - Representative images from fluorescent microscopy of hNECsstained against major basal epithelial cells markers. Scale bar, 100 μm. C - Representative images from confocal microscopy of hNOs stained KRT5 (cytoplasmic localization), TP63(nuclear localization), Muc5AC (intracellular localization) and SCGB3A2 (cytoplasmic localization) at 7 days of differentiation from hNECs. Nuclei were stained with DAPI (blue). Scale bar, 50 μm. Fig B. Binary masks predicted by the ilastik with color-coding: green – true positive, red - false positives, blue - false negative.
(DOCX)
