# Supplementary material for: A semi-automated algorithm for image analysis of respiratory organoids
Source: PLoS Comput Biol. 2025 Oct 27;21(10):e1013589. doi: 10.1371/journal.pcbi.1013589 (PMC12558486; doi:10.1371/journal.pcbi.1013589)
Supplement: S1 Text — Table A. Model performance metrics. Fig A. Characterisation of hNECs and hNOs (donor 4). A - Representative phase-contrast images of of hNECs on 1st passage and hNOs at 7 days of generation from hNECs. Scale bar, 200 μm. B - Representative images from fluorescent microscopy of hNECsstained against major basal epithelial cells markers. Scale bar, 100 μm. C - Representative images from confocal microscopy of hNOs stained KRT5 (cytoplasmic localization), TP63(nuclear localization), Muc5AC (intracellular localization) and SCGB3A2 (cytoplasmic localization) at 7 days of differentiation from hNECs. Nuclei were stained with DAPI (blue). Scale bar, 50 μm. Fig B. Binary masks predicted by the ilastik with color-coding: green – true positive, red - false positives, blue - false negative. (DOCX) [file pcbi.1013589.s001.docx]

**Supplementary Material**

Table A. Model performance metrics

|  | **U-Net model** | | | | | | **U-Net model + Cellprofiler** | | | | | |
| --- | --- | --- | --- | --- | --- | --- | --- | --- | --- | --- | --- | --- |
|  | **Accuracy** | **Precision** | **Recall** | **Specificity** | **F1_score** | **IoU** | **Accuracy** | **Precision** | **Recall** | **Specificity** | **F1_score** | **IoU** |
| **Validation set (n=41)** | | | | | | | | | | | | |
| **Mean** | 0.9967 | 0.7458 | 0.7910 | 0.9981 | 0.7613 | 0.7007 | 0.9968 | 0.7513 | 0.7727 | 0.9982 | 0.7574 | 0.7068 |
| **Std. Deviation** | 0.0061 | 0.3429 | 0.3383 | 0.0039 | 0.3364 | 0.3286 | 0.0061 | 0.3640 | 0.3601 | 0.0038 | 0.3592 | 0.3472 |
| **Median** | 0.9992 | 0.9199 | 0.9350 | 0.9994 | 0.9283 | 0.8661 | 0.9992 | 0.9349 | 0.9350 | 0.9995 | 0.9366 | 0.8807 |
| **25% Percentile** | 0.9971 | 0.6383 | 0.8705 | 0.9985 | 0.7293 | 0.5743 | 0.9973 | 0.7677 | 0.8696 | 0.9988 | 0.8049 | 0.6762 |
| **75% Percentile** | 0.9997 | 0.9663 | 0.9653 | 0.9998 | 0.9556 | 0.9150 | 0.9998 | 0.9677 | 0.9653 | 0.9999 | 0.9572 | 0.9179 |
| **Minimum** | 0.9729 | 0.0000 | 0.0000 | 0.9804 | 0.0000 | 0.0000 | 0.9724 | 0.0000 | 0.0000 | 0.9812 | 0.0000 | 0.0000 |
| **Maximum** | 1.0000 | 0.9888 | 0.9937 | 1.0000 | 0.9882 | 0.9767 | 1.0000 | 0.9888 | 0.9937 | 1.0000 | 0.9892 | 0.9787 |
| **Validation set (Subsample, n=27)** | | | | | | | | | | | | |
| **Mean** | 0.9952 | 0.9204 | 0.9504 | 0.9972 | 0.9334 | 0.8790 | 0.9953 | 0.9282 | 0.9501 | 0.9973 | 0.9373 | 0.8856 |
| **Std. Deviation** | 0.0070 | 0.0799 | 0.0301 | 0.0046 | 0.0511 | 0.0832 | 0.0071 | 0.0777 | 0.0307 | 0.0045 | 0.0494 | 0.0805 |
| **Median** | 0.9985 | 0.9446 | 0.9492 | 0.9992 | 0.9517 | 0.9079 | 0.9985 | 0.9617 | 0.9483 | 0.9993 | 0.9528 | 0.9099 |
| **25% Percentile** | 0.9931 | 0.8693 | 0.9312 | 0.9974 | 0.9198 | 0.8516 | 0.9938 | 0.9126 | 0.9325 | 0.9975 | 0.9257 | 0.8618 |
| **75% Percentile** | 0.9992 | 0.9743 | 0.9765 | 0.9997 | 0.9604 | 0.9237 | 0.9992 | 0.9743 | 0.9765 | 0.9997 | 0.9643 | 0.9311 |
| **Minimum** | 0.9729 | 0.6266 | 0.8623 | 0.9804 | 0.7497 | 0.5996 | 0.9724 | 0.6363 | 0.8604 | 0.9812 | 0.7566 | 0.6085 |
| **Maximum** | 0.9998 | 0.9888 | 0.9937 | 0.9999 | 0.9882 | 0.9767 | 0.9998 | 0.9888 | 0.9937 | 0.9999 | 0.9892 | 0.9787 |

**Immunofluorescence assay**

hNECs at the 2^nd^ passage were immunostained on KRT5 and TP63 to confirm epithelial origin. Cells were washed twice with DPBS (PanEco, Russia) and fixed with 4% paraformaldehyde (PFA) (Carl Roth, Germany) in DPBS for 10 min at 37°C. The cells were permeabilized in a cold solution of 0.1% Tween 20 (Merck, Germany) for 10 min at +4°C and washed three times with DPBS; then cells were blocked with a cold solution of 0.1% Triton X-100 (Helicon, Russia) and 0.2% bovine serum albumin (BSA) (Sigma Aldrich, USA) in DPBS for 30 min at room temperature (RT: 20°C–25°C). Primary antibodies KRT5 (ABclonal, China) and TP63 (Thermo Fisher Scientific, USA) were added and incubated for 1 h at RT. Following this, the cells were washed three times with DPBS. Then, secondary antibodies Goat anti-Rabbit IgG-AlexaFluor 488 (Absin, China) were added and incubated for 30 min at RT; the cells were washed three times with DPBS. After that, cells were stained with DAPI (Abcam, UK) and visualized using the Lionheart FX Automated Microscope.

Immunostaining of hNOs on 7 days of generation from hNECs was performed to confirm differentiation. Droplets with organoids were mechanically dislodged, centrifuged for 5 s at 6300 rpm, and fixed with 4% PFA in DPBS for 45 min at +4°C. Then, the organoids were permeabilized in a cold solution of 0.1% Tween 20 for 10 min at +4°C and centrifuged for 5 s at 6300 rpm at +4°C; the precipitate was blocked with a cold solution of 0.1% Triton X-100% and 0.2% BSA in DPBS for 15 min at +4°C. Then, a solution of primary antibodies KRT5, TP63, Muc5AC (Affinity Bioscience, China) and SCGB3A2 (ABclonal, China) was added, and the mixture was incubated overnight at +4°C. The organoids were washed twice with a solution of 0.1% Triton X-100% and 0.2% BSA in DPBS for 2 h at +4°C. Then, a solution of the secondary antibodies Goat anti-Rabbit IgG-AlexaFluor 488 was added, and the mixture was incubated overnight at +4°C. The organoids were washed twice with a solution of 0.1% Triton X-100% and 0.2% BSA in DPBS for 2 h at +4°C. After that, the organoids were stained with DAPI for 10 min at RT and subsequently centrifuged for 5 s at 6300 rpm; the pellet was resuspended in a solution of 2.5 mM fructose in 60% glycerol and incubated for 20 min at RT. The suspension was transferred onto a glass slide and covered with a cover glass; microscopy was performed on a TCS SP8 confocal laser scanning microscope (Leica Microsystems, Germany).

Fig A (A) shows images of hNECs and hNOs. The hNECs stained positively for epithelial cell markers (Fig A (B)), confirming the origin of the cells. hNOs at 7 days of differentiation from hNECs contained airway epithelial cell types: basal (KRT5+ and TP63+ cells), goblet (Muc5AC+ cells) and Club (SCGB3A2+ cells) (Fig A (С)).

*
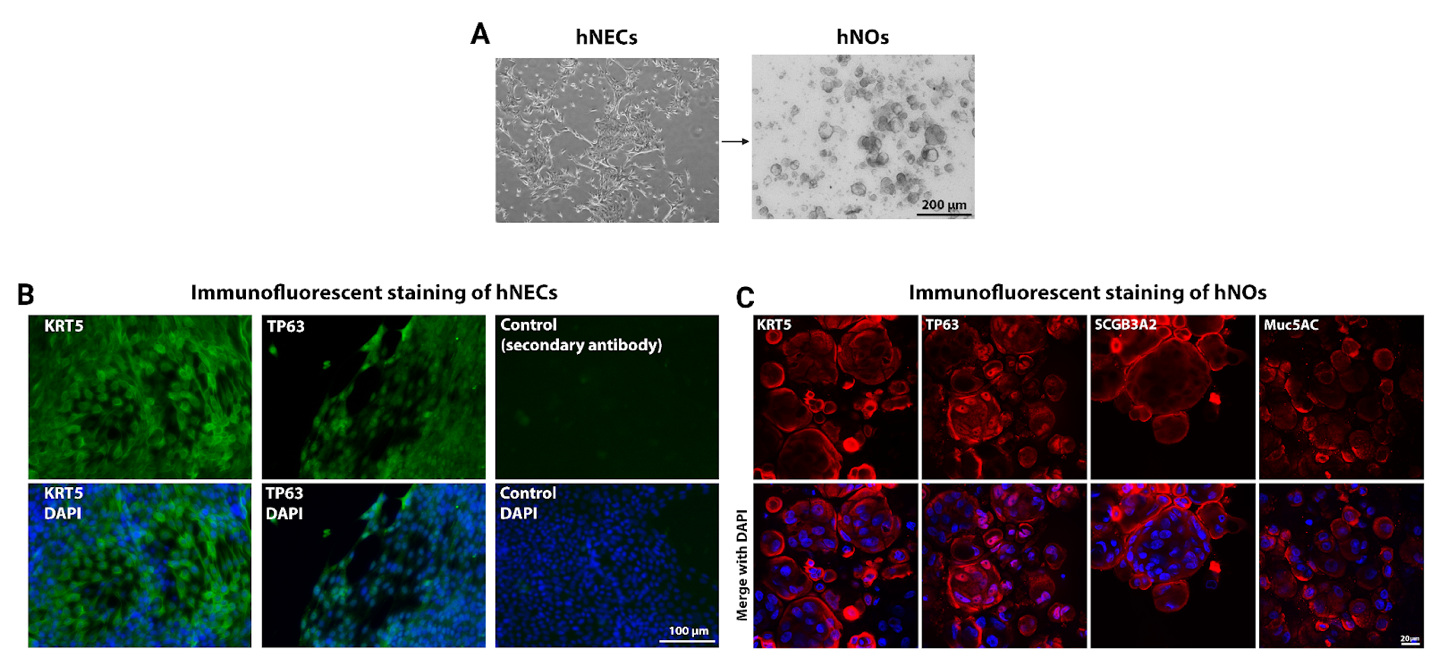
*

Fig A. Characterisation of hNECs and hNOs (donor 4). A - Representative phase-contrast images of of hNECs on 1st passage and hNOs at 7 days of generation from hNECs. Scale bar, 200 μm. B - Representative images from fluorescent microscopy of hNECs stained against major basal epithelial cells markers. Scale bar, 100 μm. C - Representative images from confocal microscopy of hNOs stained KRT5 (cytoplasmic localization), TP63 (nuclear localization), Muc5AC (intracellular localization) and SCGB3A2 (cytoplasmic localization) at 7 days of differentiation from hNECs. Nuclei were stained with DAPI (blue). Scale bar, 50 μm

**Benchmarks of model against standard method of segmentation – ilastik and CellProfiler**


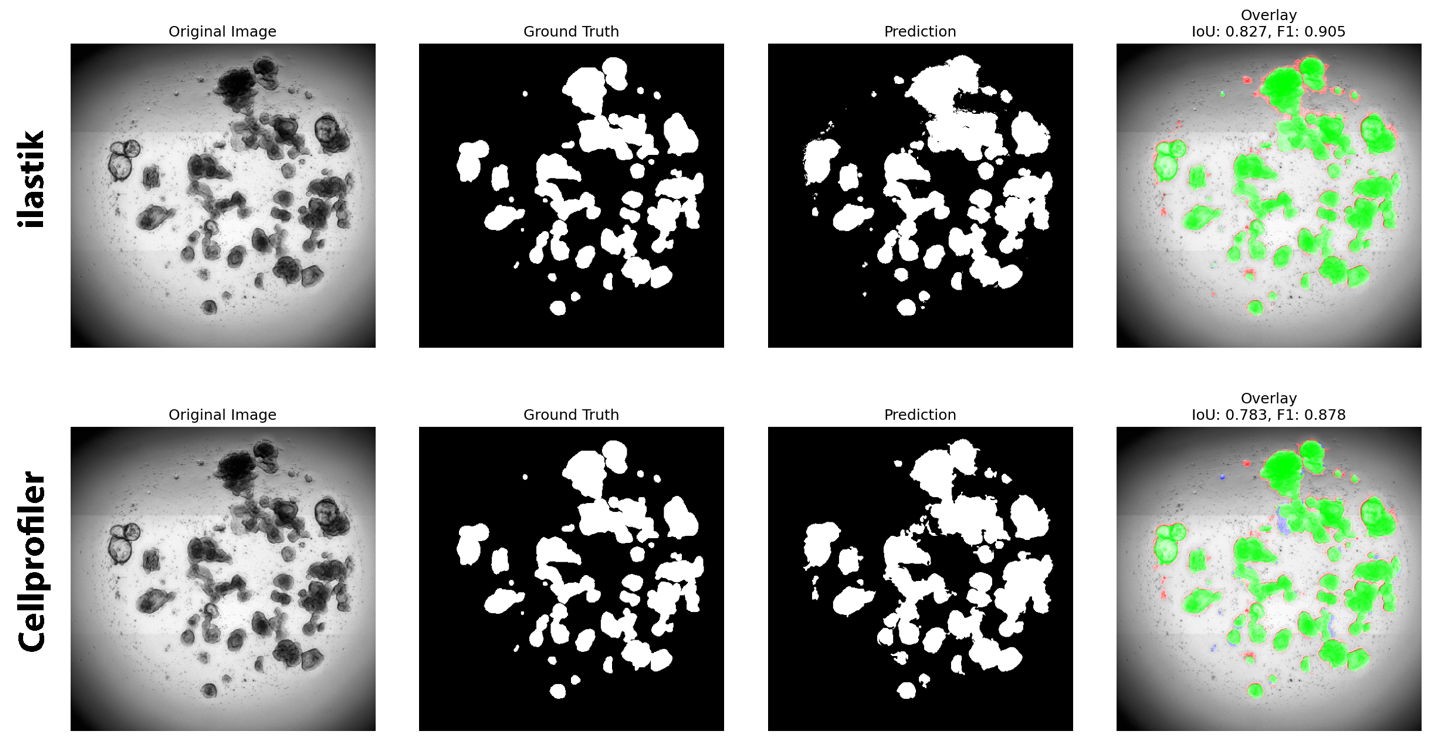


Fig B. Binary masks predicted by the ilastik and CellProfiler with color-coding: green – true positive, red - false positives, blue - false negative
